# Diagnosis of head and neck cancer by AI-based tumor-educated platelet RNA profiling of liquid biopsies

**DOI:** 10.1172/jci.insight.186680

**Published:** 2025-11-27

**Authors:** Niels E. Wondergem, Jos B. Poell, Sjors G.J.G. In ‘t Veld, Edward Post, Steven W. Mes, Myron G. Best, Wessel N. van Wieringen, Thomas Klausch, Robert J. Baatenburg de Jong, Chris H.J. Terhaard, Robert P. Takes, Johannes A. Langendijk, Irma M. Verdonck-de Leeuw, Femke Lamers, C. René Leemans, Elisabeth Bloemena, Thomas Würdinger, Ruud H. Brakenhoff

**Affiliations:** 1 Amsterdam UMC location Vrije Universiteit Amsterdam, Otolaryngology/Head and Neck Surgery, Amsterdam, Netherlands.; 2 Cancer Center Amsterdam, Head & Neck Cancer Biology & Immunology Laboratory, Amsterdam, Netherlands.; 3 Translational AI in Laboratory Medicine, Department of Laboratory Medicine, Amsterdam UMC, Amsterdam, Netherlands.; 4 Cancer Center Amsterdam, Amsterdam, Netherlands.; 5 Amsterdam Public Health, Amsterdam UMC, Vrije Universiteit, University of Amsterdam, Amsterdam, Netherlands.; 6 Amsterdam UMC location Vrije Universiteit Amsterdam, Neurosurgery, Amsterdam, Netherlands.; 7 Brain Tumor Center Amsterdam, Amsterdam, Netherlands.; 8 Amsterdam UMC location Vrije Universiteit Amsterdam, Epidemiology and Data Science, Amsterdam Public Health Research Institute, Amsterdam, Netherlands.; 9 Department of Mathematics, Vrije Universiteit Amsterdam, Amsterdam, Netherlands.; 10 Department of Otolaryngology and Head and Neck Surgery, Erasmus University Medical Center, Rotterdam, Rotterdam, Netherlands.; 11 Department of Radiotherapy, University Medical Center Utrecht, Utrecht, Netherlands.; 12 Department of Otolaryngology and Head and Neck Surgery, Radboud University Medical Center, Nijmegen, Netherlands.; 13 Department of Radiation Oncology, University Medical Center Groningen, Groningen, Netherlands.; 14 Department of Clinical, Neuro- and Developmental Psychology, Amsterdam Public Health Research Institute, Vrije Universiteit Amsterdam, Netherlands.; 15 Amsterdam UMC location Vrije Universiteit Amsterdam, Psychiatry, Neuroscience Campus Amsterdam and Amsterdam Public Health Research Institute, Amsterdam, Netherlands.; 16 Amsterdam UMC location Vrije Universiteit Amsterdam, Pathology, Amsterdam, Netherlands.; 17 Amsterdam UMC and Academic Centre for Dentistry Amsterdam (ACTA), Vrije Universiteit Amsterdam, Department of Oral and Maxillofacial Surgery/Pathology, Cancer Center Amsterdam, Amsterdam, Netherlands.

**Keywords:** Genetics, Oncology, Diagnostics, Head and neck cancer, Platelets

## Abstract

Over 95% of head and neck cancers are squamous cell carcinoma (HNSCC). HNSCC is mostly diagnosed late, causing a poor prognosis despite the application of invasive treatment protocols. Tumor-educated platelets (TEPs) have been shown to hold promise as a molecular tool for early cancer diagnosis. We sequenced platelet mRNA isolated from blood of 101 patients with HNSCC and 101 propensity-score matched noncancer controls. Two independent machine learning classification strategies were employed using a training and validation approach to identify a cancer predictor: a particle swarm optimized support vector machine (PSO-SVM) and a least absolute shrinkage and selection operator (LASSO) logistic regression model. The best performing PSO-SVM predictor consisted of 245 platelet transcripts and reached a maximum area under the curve (AUC) of 0.87. For the LASSO-based prediction model, 1,198 mRNAs were selected, resulting in a median AUC of 0.84, independent of HPV status. Our data show that TEP RNA classification by different AI tools is promising in the diagnosis of HNSCC.

## Abstract 2

RNA expression profiling of tumor-educated platelets is highly accurate in detecting head and neck cancer, and could aid head and neck cancer diagnostics.

## Introduction

Head and neck squamous cell carcinoma (HNSCC) arises in the mucosal linings of the upper respiratory and digestive tract, comprising the oral cavity, oropharynx, hypopharynx, and larynx. It ranks among the most prevalent cancers, affecting more than 890,000 patients annually and resulting in over 450,000 deaths in 2022 (1). Risk factors for developing HNSCC are tobacco and alcohol consumption and, particularly in the oropharynx, persistent infection with high-risk human papillomavirus (HPV) (2).

Patients with early-stage disease undergo local single modality treatment and have a 90% 5-year overall survival (OS) with good quality of life. However, most patients present with locally advanced disease and are treated with definitive chemoradiotherapy or extensive surgery with reconstruction combined with postoperative (chemo)radiotherapy, which affect quality of life while the 5-year OS remains 40%–50% (3). Despite a variety of advances in treatment modalities, the survival of HNSCC remains disappointing. Hence, early diagnosis is key to improve both prognosis of HNSCC and the quality of life of treated patients, as less invasive treatment protocols can be applied (4). Late diagnosis is often due to the mild initial symptoms and delay in recognition by primary care physicians, as early-stage HNSCC can be challenging to identify (5).

In this setting, liquid biopsies have gained increasing interest as a novel, minimally invasive means of early cancer diagnostics through the detection of biomarkers in body fluids like blood, urine, and saliva. Samples from these compartments are easy to obtain and could eliminate the need for a tumor biopsy and its inherent risk of complications. Biomarkers under investigation include circulating tumor cells (CTCs), extracellular vesicles (EVs), circulating cell-free DNA (cfDNA), circulating cell-free RNA (cfRNA) and tumor-educated platelets (TEPs) (6). Their relative abundance and straightforward isolation make TEPs a highly interesting biomarker for minimally invasive cancer diagnosis (7, 8).

Platelets are traditionally known for their function in hemostasis and wound healing, but they have more recently been recognized as protumorigenic entities in the tumor microenvironment (TME) (7). Platelets have been shown to induce prosurvival and proangiogenic signaling and participate in the epithelial-mesenchymal transition (EMT) of tumor cells, facilitating invasiveness and metastasis (9). Moreover, platelet aggregation around CTCs provides shielding from immune surveillance and reduces mechanical stress endured in the bloodstream (10). Tumor cells can induce differential splicing of platelet pre–messenger RNA (pre-mRNA) in their interaction with platelets. Additionally, platelets are able to ingest tumor-derived biomolecules such as mRNA (11, 12). These processes are proposed to constitute the tumor education of platelets, now referred to as TEPs, and provide them with a changed mRNA repertoire which can be exploited for cancer diagnostics, as shown by Best et al. (13). By performing RNA-Seq on platelets and employing a self-learning support vector machine (SVM) algorithm, they were able to discriminate patients with cancer from healthy controls with 96% accuracy (13). Results were validated in multiple follow-up studies and by other groups in various cancer types (14–19).

Crucial in the evaluation of biomarkers in cancer diagnostics, including TEPs, is the use of a well-matched control group. Particularly for patients with HNSCC, increased age, smoking habits, and alcohol consumption might affect TEP expression patterns, highlighting the importance of well-matched control groups (20, 21). Previously, a comprehensive protocol for platelet RNA-based diagnostic algorithm development was published, providing a detailed step-by-step wet and dry lab manual to be exploited by scientists in their field of interest (22). In this study, we applied this protocol and evaluated an alternative feature selection and classification strategy as compared with the original protocol, and investigated the applicability of TEPs as diagnostics in a cohort of patients with HNSCC and propensity-score matched noncancer controls.

## Results

RNA of all 214 samples was sequenced with a mean read count of ~10 million reads per sample. After data quality control steps filtering out low-abundance reads (22), 5,546 of 57,736 annotated RNA transcripts remained, which were used for subsequent classification model development. In total, 6 samples had to be excluded because of poor data quality, indicating that 208 of 214 (97%) were diagnostic. To maintain a propensity-score matched 1:1 dataset, the corresponding matched sample for each sample excluded during quality control had to be excluded as well, resulting in a cohort of 202 samples containing 101 patients with HNSCC and 101 matched cancer-free controls (Supplemental Figure 1; supplemental material available online with this article; https://doi.org/10.1172/jci.insight.186680DS1). The clinical characteristics are depicted in Table 1. Variables — age, smoking status, packyears, and alcohol consumption — were evenly distributed among groups as a result of matching. Cases included 70% males compared with 35% in the control group (*P* < 0.001). This was a consequence of the design of the NET-QUBIC study where patients’ spouses were included as cancer-free controls. Since most patients with HNSCC are males, most controls were female. As we could not correct this by matching, it was corrected for by the Remove Unwanted Variation (RUV) function as described in the Methods section.

### PSO-SVM classification.

**Figure 1:**
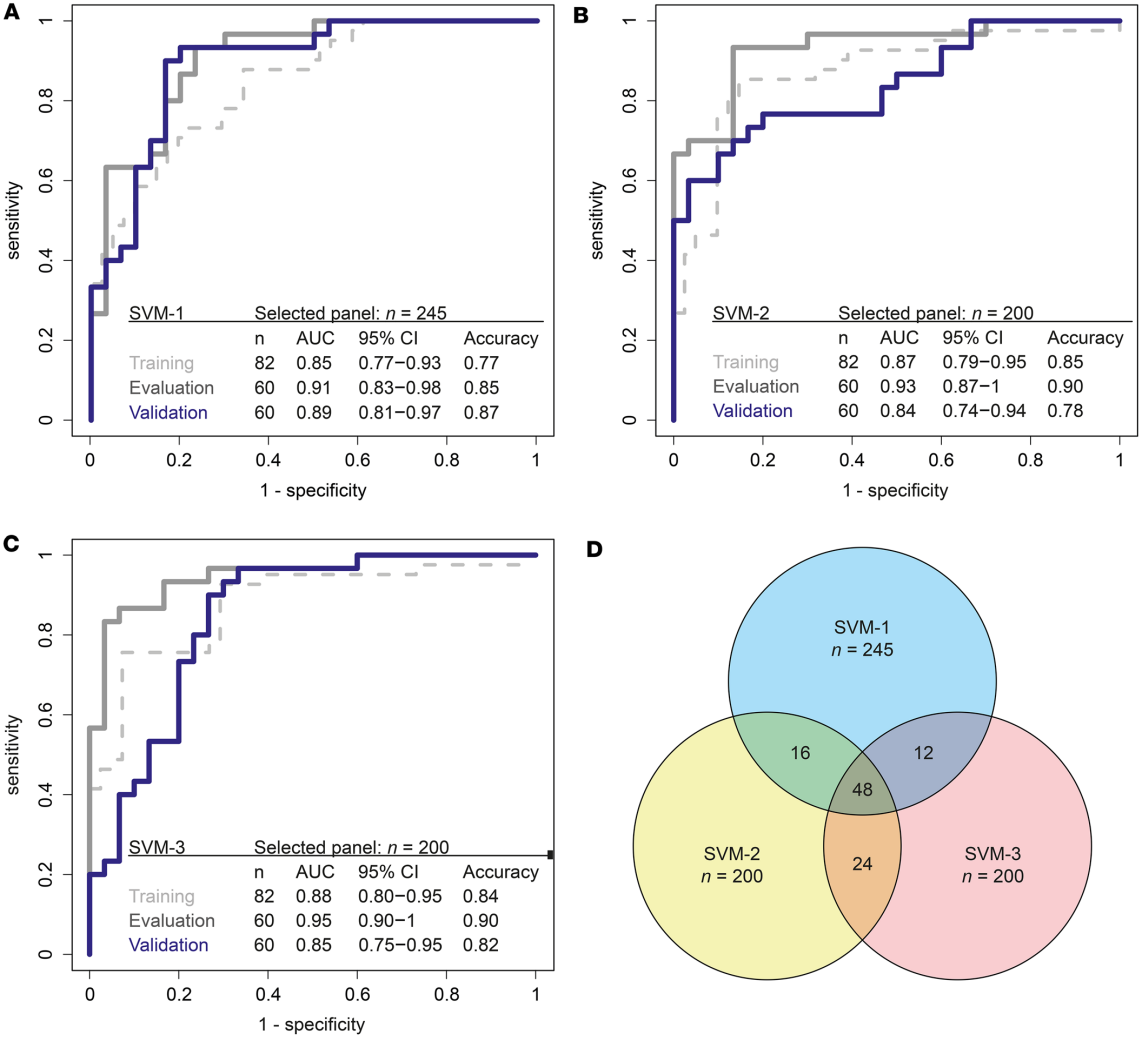
Performance of learned SVMs. (**A**–**C**) ROC curves and summary of performance of training, evaluation, and validation series for learned SVMs 1–3 on the dataset of HNSCC (*n* = 101) and matched controls (*n* = 101). (**D**) Venn diagram showing overlap between selected biomarker panels of learned SVMs 1–3. Training set is indicated by a dashed light grey line, evaluation set by a solid grey line and validation set by a solid blue line. ROC, receiver operator characteristic; SVM, support vector machine; AUC, area under the curve; CI, confidence interval.

First, we identified the most predictive TEP RNA transcripts, using particle swarm optimization (PSO) to identify the RNAs resulting in the best unsupervised hierarchical clustering. Out of 5,546 differentially expressed transcripts, 941 transcripts identified by a PSO threshold of FDR < 0.0018 were found to be particularly useful for the discrimination between cases and controls (Fisher exact test *P* < 0.0001; Supplemental Figure 2). These 941 transcripts were forwarded to the SVM algorithm. For training of the SVM, paired samples were randomly divided into a training set (40%, *n =* 41 HNSCC cases and *n =* 41 matched controls), evaluation set (30%, *n =* 30 HNSCC cases and *n =* 30 matched controls), and validation set (30%, *n =* 30 HNSCC cases and *n =* 30 matched controls). A general caveat in machine learning algorithms is that results are highly dependent on the composition of the training, evaluation, and validation sets. To evaluate the effect of sample distribution on model performance and selected biomarker panels, 3 SVM classification models were developed with 3 different, random distributions. Subsets were checked for significant differences in age, smoking, smoking pack years, and alcohol use to ensure that no bias was introduced by chance (Supplemental Figure 3). The first analysis (SVM-1) had 245 transcripts in the predictor and resulted in an area under the curve (AUC) of 0.87 (95% CI, 0.81–0.97) and 87% accuracy. The second analysis (SVM-2) resulted in 200 transcripts with an AUC of 0.84 (95% CI, 0.74–0.94) and 78% accuracy. The third SVM analysis (SVM-3) resulted in 200 transcripts with an AUC of 0.85 (95% CI, 0.75–0.95) and 82% accuracy (Figure 1, A–C). A Venn diagram of the selected transcripts for SVM-1, SVM-2, and SVM-3 showed an overlap of 48 RNA transcripts between the 3 analyses (Figure 1D and Supplemental Table 1).

### LASSO classification.

**Figure 2:**
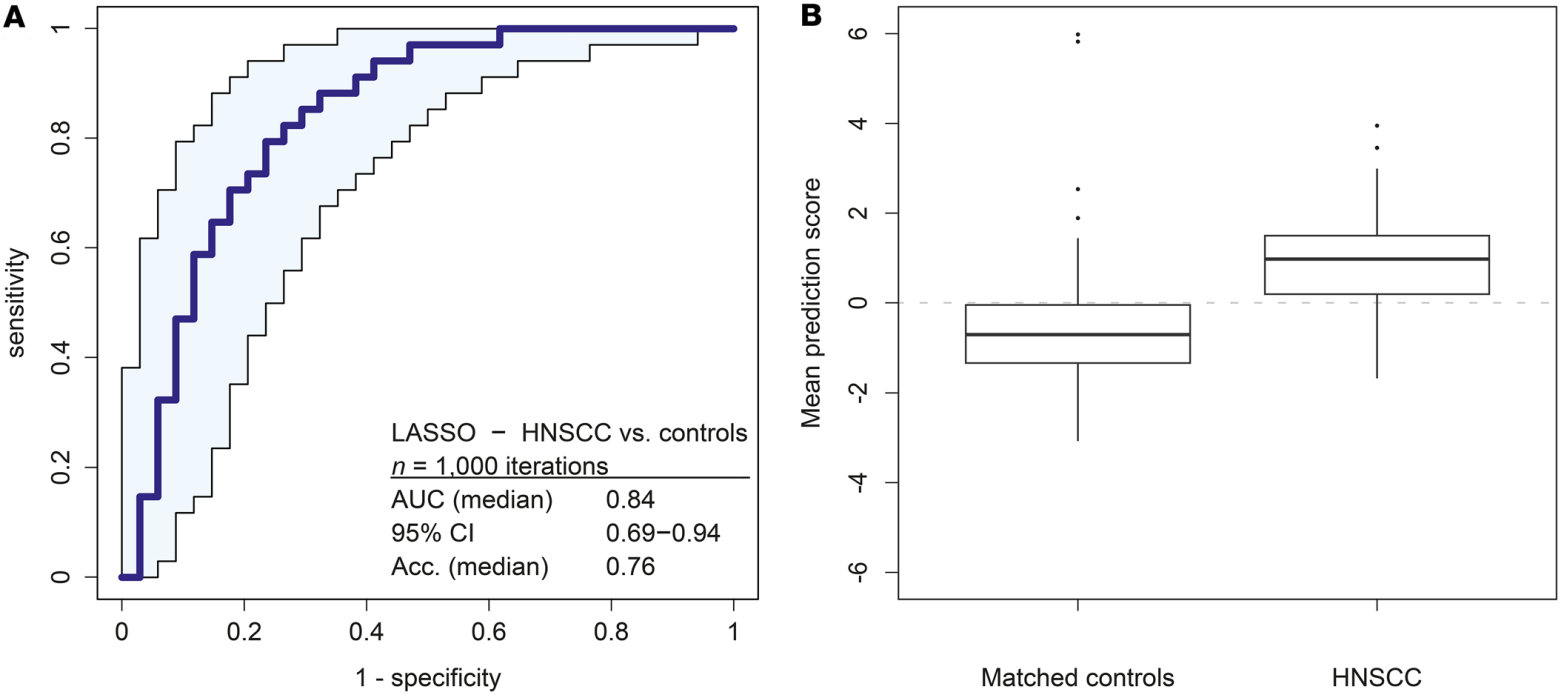
Performance of LASSO models. (**A**) ROC curve and summary of performance of validation series for 1,000 LASSO models on the dataset of HNSCC (*n* = 101) and matched controls (*n* = 101). In total, 1,198 genes were selected and used for the prediction models. (**B**) Box plot of the mean prediction scores of matched controls and HNSCC samples in 1,000 LASSO models. ROC, receiver operator characteristic; LASSO, least absolute shrinkage and selection operator; HNSCC, head and neck squamous cell carcinoma; AUC, area under the curve; CI, confidence interval; Acc., accuracy.

The 3 SVM models indicated that TEP profiling was highly accurate to distinguish patients with HNSCC from matched controls. Still, the considerable variation in selected transcripts affects extrapolation to the clinical setting and prompted an alternative analysis method using a least absolute shrinkage and selection operator (LASSO). For LASSO classification, samples were randomly divided into a set for training and internal validation (66%, *n =* 67 HNSCC cases and *n =* 67 matched controls) and a set for external validation (34%, *n =* 34 HNSCC cases and *n =* 34 matched controls). Sample selection for the training and validation sets and subsequent classifier development was iterated 1,000 times, resulting in 1,000 classification models. These models reached a median AUC of 0.84 (95% CI, 0.69–0.94) and a median accuracy of 76% (Figure 2). Accuracy was dependent of disease stage: samples of early stage (TNM disease stage I/II) and advanced stage (TNM disease stage III/IV) tumors showed an detection accuracy of 68% and 86%, respectively (Table 2).

A total of 1,198 transcripts had a nonzero coefficient in at least 1 LASSO regression model. To investigate which of the 1,198 RNA transcripts were most contributing to HNSCC classification, we compared the observed frequency of transcripts with a nonzero coefficient in all selected 1,000 transcript panels to the frequency expected by random selection. In total, 316 transcripts were significantly overrepresented and are determining the prediction (FDR < 0.001, corresponding to observed frequency of ≥ 16; Supplemental Figure 4). The most predictive transcript was *AKT1*, which was selected in 950 of 1,000 iterations. Overexpression of *AKT1* in HNSCC has been reported, and mutational activation of the *PI3K/AKT/mTOR* pathway is known to increase tumor cell survival (23–25). STRING analysis of all 316 transcripts showed enrichment for 41 biological processes (FDR < 0.05; Supplemental Table 2), which mainly comprised metabolic processes.

### Evaluation of HPV infection on TEP RNA signatures.

Oropharyngeal SCC (OPSCC) is either caused by exogenous carcinogens or HPV infection. It has been shown that platelets can take up genetic material from other cells, including mutation-harboring fragments from tumor cells (26). We hypothesized that transcripts originating from HPV could contribute to the discriminative power of TEP RNA between cases and controls. Therefore, the sequenced reads of all patients with OPSCC were mapped against the HPV genome, which yielded no hits in both the HPV^+^ and HPV^–^ groups, indicating that HPV-derived RNA transcripts could not be detected in TEP samples of OPSCC patients, at least not with this sequencing coverage. Of note, like human RNA, HPV RNA has a poly-A tail and is expected to be amplified during sample work-up, if present. To evaluate the potential effect of HPV infection on TEP RNA signatures in OPSCC, we compared the LASSO classification accuracy and found that HPV^+^ and HPV^–^ patients with OPSCC were classified with fairly comparable accuracies of 80% and 86%, respectively (Table 2), suggesting that HPV status is not of key relevance in the current study design. A LASSO based model trained to predict HPV-status resulted in a median AUC of 0.62 (95% CI, 0.35–0.89) and median accuracy of 58% after 1,000 iterations (Supplemental Figure 5), confirming that TEP profiles are not HPV specific. To assess the possibility that this poor prediction was due to the limited sample size, we developed a LASSO model on the primary cohort, randomly downsized to a sample size comparable with the OPSCC subcohort (*n* = 60; 30 HNSCC and 30 matched controls). We were still able to reach a median AUC of 0.78 (95% CI, 0.53–0.89) with a median accuracy of 72%. These results indicate that an HPV-specific effect on TEP signatures, if any, is limited when compared with the presence or absence of HNSCC and undetectable with our current classification methods.

## Discussion

This study aimed to investigate TEP RNA profiles as a biomarker for the diagnosis of HNSCC. We analyzed a well-annotated cohort of 101 HNSCC cases containing all HNSCC subsites and all stages, propensity score matched to an equal number of noncancer controls. Matched variables included age and the classical risk factors for developing HNSCC: smoking status, smoking pack years, and alcohol use. Notably, age and smoking have previously been described to be of significant statistical contribution to TEP RNA classifiers in nonsmall cell lung cancer, underlining the importance of matching to account for the possible confounding effects of these clinical variables (14).

From a technical perspective, the use of SVM algorithms for classification tasks is well established in various fields, including cancer genomics. In this study, we identified a significant limitation: the selected gene panels may heavily depend on the distribution of samples between the training, evaluation, and validation sets. This is exemplified by the 3 SVM algorithms, which yielded different gene panels with small overlap. This could be further exaggerated by the funneling effect of PSO, which selects features based on previous results, potentially pushing, especially smaller training sets, into a specific gene panel selection. Overfitting of the model remains a critical pitfall, especially with smaller datasets. Despite this high variance in feature panel, the performance of all models was excellent, with AUCs ranging from 0.84 to 0.89, indicating the power of the TEP profiling approach. We sought to overcome the effect of sample distribution on gene panel selection by subjecting our data to a LASSO classification algorithm, enabling us to merge the training and evaluation sets into a larger training set. LASSO requires less computational power, allowing the algorithm to be iterated 1,000 times in a much shorter time with fewer cores compared with PSO-SVM. Each iteration used a different random sample distribution, which resulted in the development of 1,000 LASSO classification models reaching a median AUC of 0.84. In all LASSO iterations, 1,198 transcripts were selected, of which 316 had the most discriminating potential in the overall cohort.

From a biological perspective, it is remarkable that there was no difference between HPV^+^ and HPV^–^ tumors, and TEP profiles were able to predict the presence of HNSCC equally well for HPV^+^ and HPV^–^ cases, a perception that certainly supports clinical translation. The classification performance was affected by disease stage, which is a common finding within liquid biopsy prediction models (27). Tumor volumes have an effect on the changes in the blood, both for circulating tumor DNA and for TEPs. The precise mechanisms by which tumors affect platelets, leading to their activation, is unknown. This could relate to aberrant metabolism, hypoxia, changes in exosomes, or direct tumor-platelet interaction (28). The observation that the LASSO-selected and best predicting transcripts relate to metabolic processes might be indicative of hypoxia or metabolic influences. Still, future in vitro experiments would be required to study this in more detail. Irrespective of the precise molecular background, the HNSCC diagnostic performance of TEP profiling is encouraging (19). In suspected cases, blood samples can be drawn in primary care, where the diagnosis can be made, and patients can be referred. Not only might screening approaches in high-risk populations be of value, but disease monitoring is also an option. Ideally, liquid biopsy analyses would facilitate detection of relapses at the earliest possible stage, which would improve the possibilities for curative salvage treatment and patient survival. In patients with glioblastoma, this potential has already been demonstrated: TEP tumor scores reflected glioblastoma burden and could be used to differentiate between true and false positive progression (16).

A strength of the current study is that controls were selected from a pool of spouses, meaning that blood samples were drawn and processed at the exact moment in the same laboratory, reducing the potential confounding effect of laboratories on the resulting platelet RNA profiles. A limitation, as a consequence of the study design, is that we could not match the data for sex and, therefore, computationally had to correct the data for sex by employing RUV. Ideally, the control group would be sex matched, eliminating the need for bioinformatic corrections. Secondly, as a consequence of the inclusion protocol, 35% of cases were early stage HNSCC. While most clinical advantage would be gained in early detection of HNSCC, requiring early-stage samples for optimal classification algorithm development, our cohort reflected the distribution of disease stage at primary clinical presentation. Follow-up studies focusing on early-stage HNSCC solely should further explore the use of TEPs for early-stage detection. Finally, when studying the role of HPV, our subcohort of OPSCC was relatively small, which could have affected model performance and underestimation of the model.

In conclusion, we successfully analyzed platelet RNA from a matched cohort. We showed that 2 independent classification strategies were able to differentiate patients with HNSCC from matched cancer-free controls with promising accuracy. For future clinical implementation including monitoring for recurrent disease, early-stage classification development and large-scale longitudinal series are required.

## Methods

### Sex as a biological variable.

Our study cohort included male and female participants. Data were corrected for sex effect.

### Study participants.

The study cohort included platelet samples from 107 patients with HNSCC and 107 cancer-free controls propensity score matched for age, smoking status, smoking pack years, and alcohol use, variables known to influence platelet profiles (14, 20, 21). All patients had a histologically confirmed diagnosis of primary HNSCC and were treatment-naive at the time of sample collection. Patients were staged according to the seventh edition of the American Joint Committee on Cancer staging manual, as patients were enrolled while the seventh edition was in use. The control pool consisted of patients’ spouses to minimize confounding variables that could affect classification results, such as the time of blood collection, on-bench storage time before processing, reagent, and sequencing batches. For HPV analyses, we compiled a cohort of patients with OPSCC from the matched cohort plus a surplus of 15 patients with OPSCC that had been TEP profiled but could not be included in the matched case-control cohort due to lack of a matched control. This resulted in a total of 55 patients with OPSCC, of which *n =* 25 were HPV^–^ and *n =* 30 were HPV^+^. Samples were obtained from the Head and Neck Cancer Collection (HNcol) study and the Netherlands Quality of life and Biomedical Cohort (NET-QUBIC) (29).

### Matching.

The *MatchIt* (v4.5.0) package was used for propensity score matching (30). Covariates age, smoking status, smoking packyears, and alcohol use were included in a logistic regression model, and controls were matched to cases in a 1:1 ratio using the nearest-neighbor method with a caliper of 0.2. There remained a sex imbalance that was corrected for in the analyses.

### Sample work-up and data processing.

Blood sampling, platelet RNA isolation, library preparation, sequencing, and data processing were performed as published by Best et al. (22). In brief, raw sequencing data were prepared for mapping using *Trimmomatic* (v0.22) and mapped using *STAR* (v2.3.0) (31, 32). Picard tools was used for adding read groups and *SAMtools* (v1.15.1) for .sam-to-.bam file conversion and data sorting, after which intron-spanning reads were selected to exclude reads from contaminating DNA molecules. Reads were summarized using *HTSeq* (v0.6.1) (33). To reduce noise, only those transcripts with sufficient read coverage (>30 reads in >90% of the samples) were selected for algorithm development. Samples with < 750 transcripts were excluded, as these would not have reads for all transcripts selected for the algorithm (*n* = 3). Data were subsequently subjected to trimmed mean of M values (TMM) normalization, and RUV correction to correct for library size and sex (34, 35). Samples with suboptimal coverage (<1 mean reads per gene) were also excluded (*n* = 3).

### Classification model development.

Classification models were developed using either a SVM or a LASSO approach. For the learned SVM, differential expression analysis was performed to compare RNA expression levels between HNSCC samples and matched cancer-free controls using 2-way ANOVA, and the total number of features was reduced by selecting the most contributive transcripts on basis of their FDR. Subsequently, the reduced biomarker panel was used for the SVM classification algorithm. The SVM was trained on a training series (40% of samples), after which performance was evaluated in an independent evaluation series (30% of samples). This process was repeated, allowing PSO of SVM parameters and selection of the most contributive transcripts, optimizing the AUC of the evaluation series after each iteration. The settings yielding the highest AUC in the evaluation set were subsequently locked and used to validate the classifier in an independent validation series (remaining 30% of samples).

For LASSO, the R-package *penalized* (v0.9-52) was used (36). A training/internal validation series (66% of samples) was selected to perform logistic regression to estimate regression coefficients for all transcripts that remained after preprocessing, but without FDR threshold preselection. LASSO penalizes the regression coefficients toward zero, effectively removing transcripts least associated with the outcome of interest and thereby reducing the biomarker panel. The penalty is controlled by tuning parameter λ, which was optimized for each iteration toward a maximum AUC using a 10-fold cross validation. All transcripts with a nonzero coefficient were subsequently used for the prediction of an external validation series (remaining 34% of samples).

### STRING analysis.

STRING v.12 (www.string-db.org) analysis was performed to identify enrichment for biological processes among the genes most contributive to TEP based HNSCC classification models (37).

### Statistics.

All statistical analyses were performed using R (version 4.4.3; http://www.Rproject.org). For comparison of group characteristics, 2-tailed parametric *t* tests were used, and the Wilcoxon Mann-Whitney test when variables were not normally distributed. Two-way ANOVA was used for differential expression analysis. *P* < 0.05 was considered statistically significant. SVM and LASSO model performance were summarized in a receiver operating characteristic (ROC) curve.

### Study approval.

The NET-QUBIC and HNcol studies were approved by the IRB of Amsterdam UMC, location Vrije Universiteit Medical Center: NET-QUBIC 2013.301(A2018.307)-NL45051.029.13; HNcol 2008/71- NL22230.029.08. All patients provided written informed consent.

### Data availability.

Clinical information on patients and controls and the raw sequencing data are available through the NET-QUBIC data repository upon request through https://data.onderzoek.io/kubus/ A step-by-step wet and dry lab protocol was previously published by Best et al. (22). Additional R code to perform LASSO classification is available through https://github.com/nielsevert/TEP; commit ID 8c59a51. Supporting data values for all figures are provided in the Supporting Data Values file.

## Author contributions

Conceptualization was contributed by EB, TW, and RHB. Methodology was contributed by TW, SGJGIV, MGB, EP, WNV, and TK. Formal analysis was contributed by NEW, JBP, SWM, WNV, and TK. Resources were contributed by RJB, CHJT, RPT, JAL, IMVL, FL, and CRL. Writing of the original draft was contributed by NEW and RHB. Review and editing were contributed by NEW, JBP, SGJGIV, EP, SWM, MGB, WNV, TK, RJB, CHJT, RPT, JAL, IMVL, FL, CRL, EB, TW, and RBR. Visualization was contributed by NEW. Supervision was contributed by RHB. Project administration was contributed by NEW and RBR. Funding acquisition was contributed by EB, TW, and RHB.

## Funding support

The study was funded by the Hanarth Fund. The NET-QUBIC study was funded by the Dutch Cancer Society (grant no. VU 2013–5930). The funding bodies did not participate in study design or interpretation of the data.

## Supplementary Material

Supplemental data

Supporting data values

## Figures and Tables

**Table 1:**
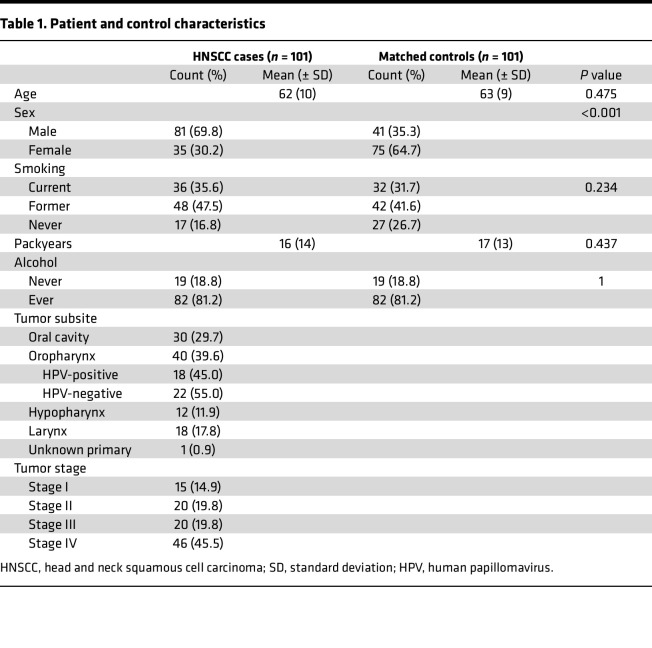
Patient and control characteristics

**Table 2:**
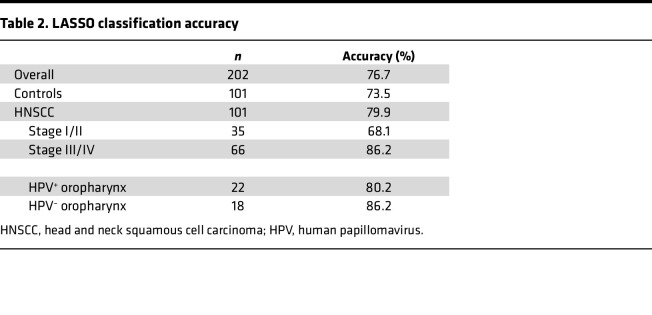
LASSO classification accuracy

## Version 1 11/27/2025

In-Press Preview

## Version 2 01/23/2026

Electronic publication

## Version 3 03/31/2026

Correction to an author's name
