# Supplementary material for: Diagnosis of head and neck cancer by AI-based tumor-educated platelet RNA profiling of liquid biopsies
Source: JCI Insight. 2025 Nov 27;11(2):e186680. doi: 10.1172/jci.insight.186680 (PMC12892895; doi:10.1172/jci.insight.186680)
Supplement: Supplemental data [file jciinsight-11-186680-s005.pdf]

Supplemental Materials

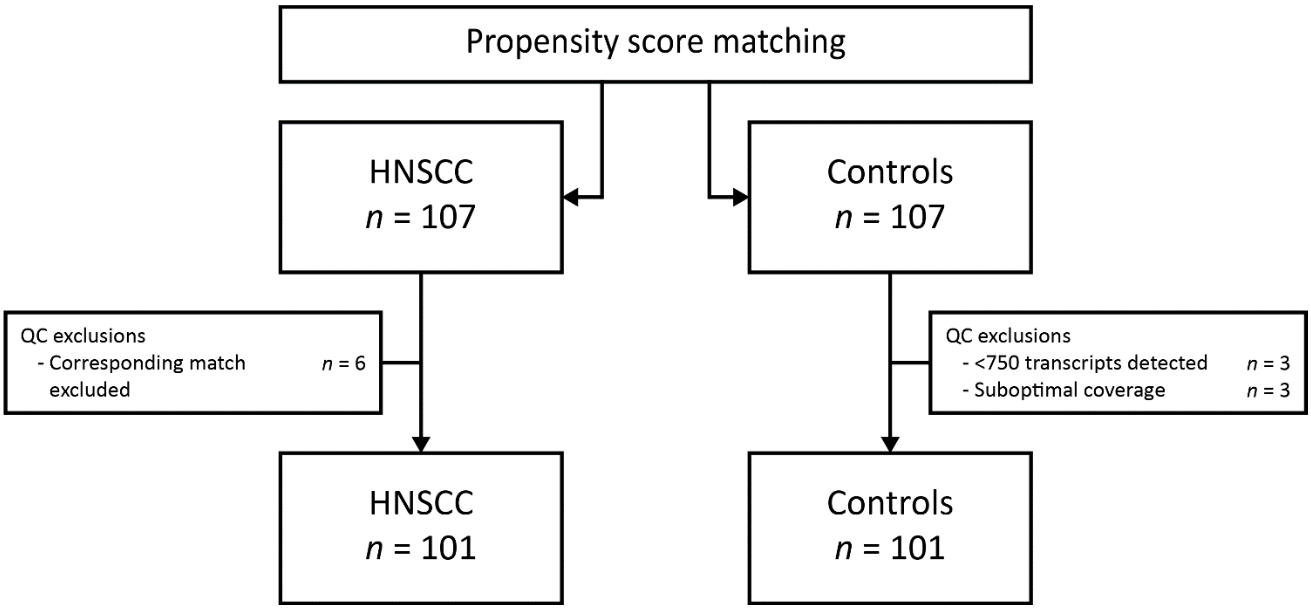

Supplemental Figure 1 Flow-chart indicating samples excluded at quality control steps. HNSCC = head and neck squamous cell carcinoma; QC = quality control.

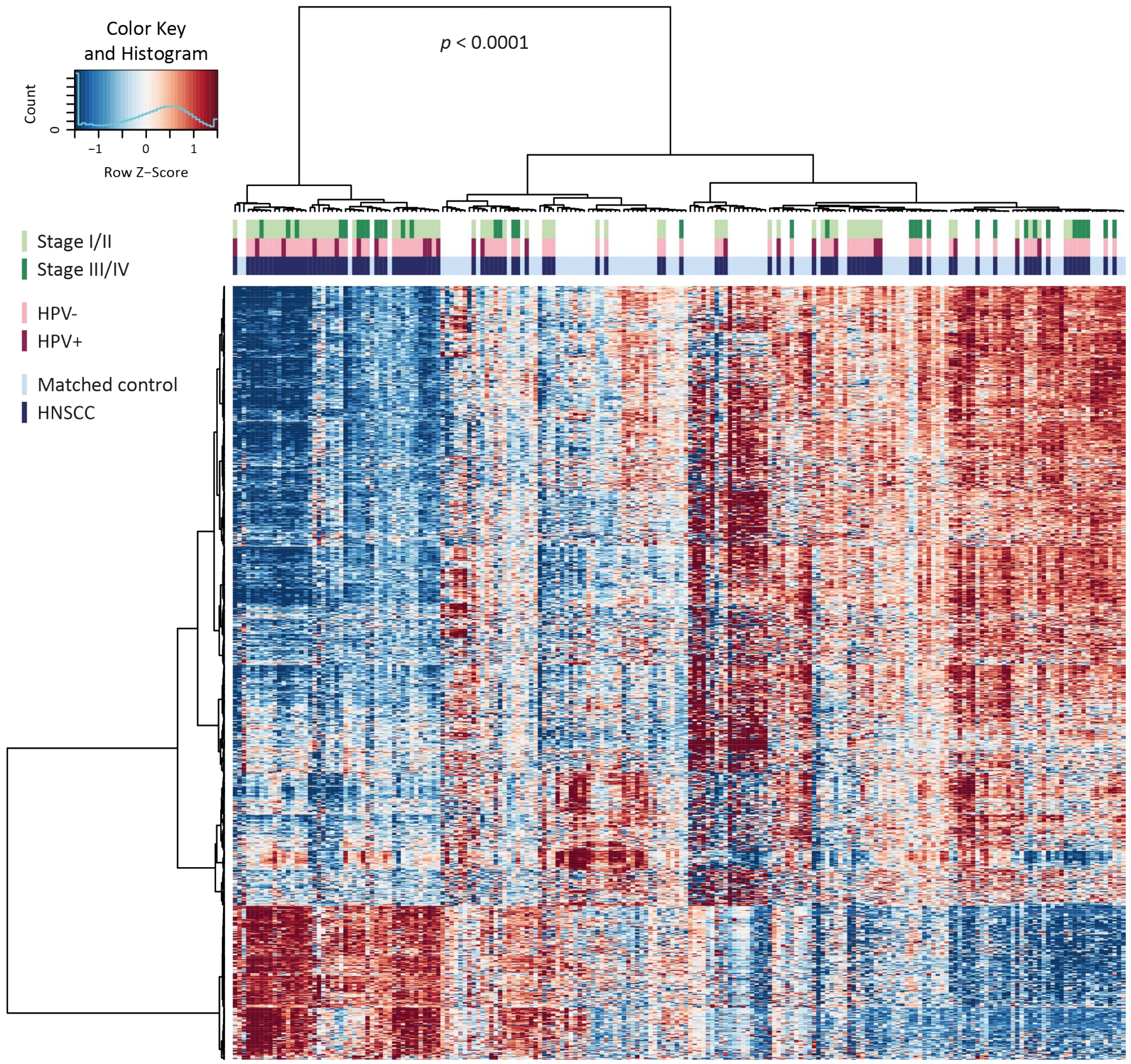

Supplemental Figure 2 Heatmap showing unsupervised clustering of 941 differentially expressed RNA transcripts (PSO selected FDR < 0.0018) between HNSCC (*n* = 101) and matched controls (*n* = 101) individuals. The rows indicate RNA transcripts, the columns indicate samples. The Z-score transformed RNA expression values are represented by colour intensity: red represents high expression, blue represents low expression. Unsupervised hierarchical clustering showed significant clustering of a subgroup of 42 HNSCC cases ($p < 0.0001$). FDR = false discovery rate; HNSCC = head and neck squamous cell carcinoma; HPV = human papillomavirus.

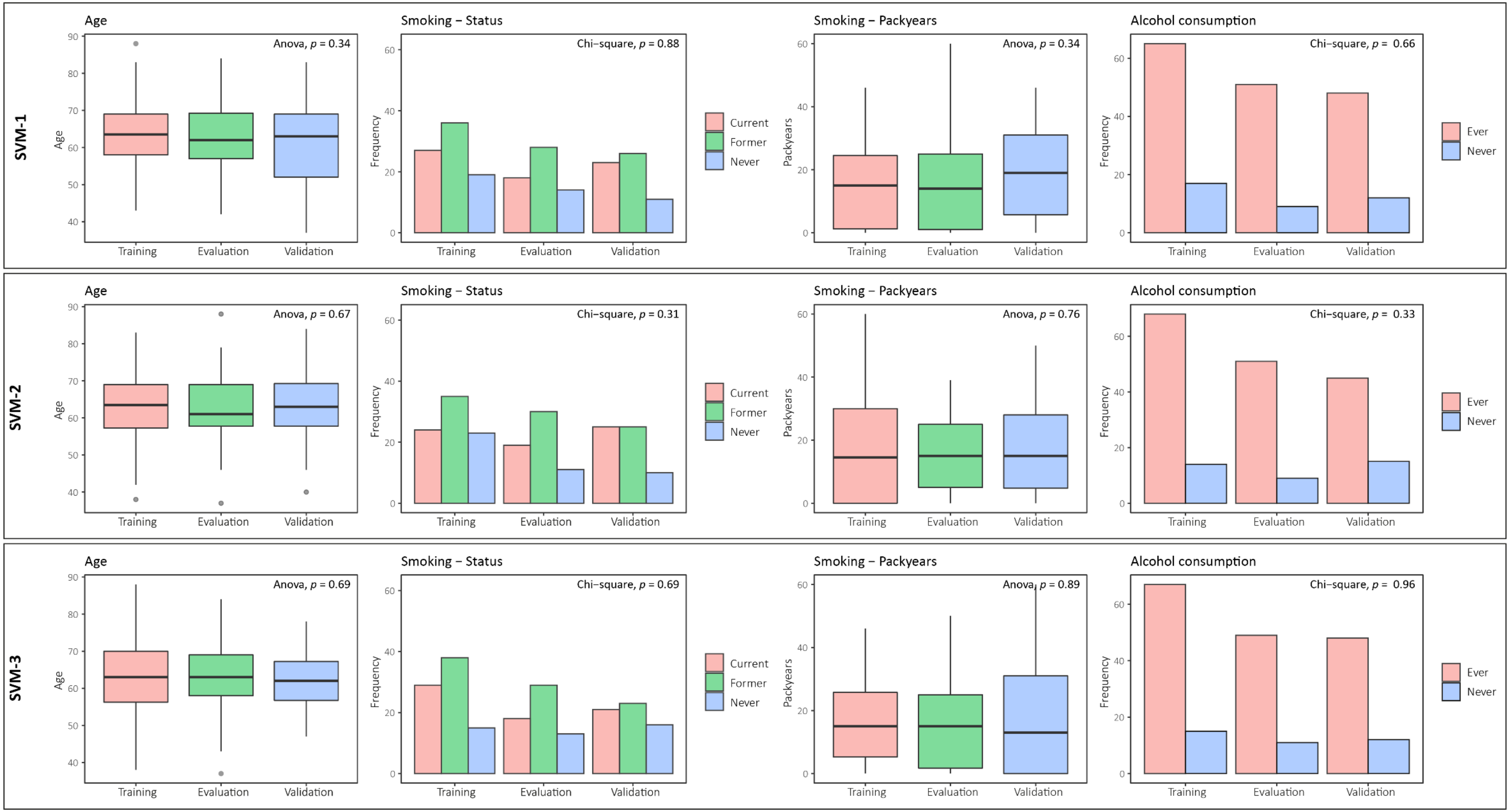

Supplemental Figure 3 Boxplots for variables age and smoking packyears and bargraphs for variables smoking status and alcohol consumption showing the distribution of variables over the training (*n* = 41 HNSCC, *n* = 41 matched controls), evaluation (*n* = 30 HNSCC, *n* = 30 matched controls), and validation (*n* = 30 HNSCC, *n* = 30 matched controls) sets for SVM-1, SVM-2 and SVM-3. SVM = support vector machine.

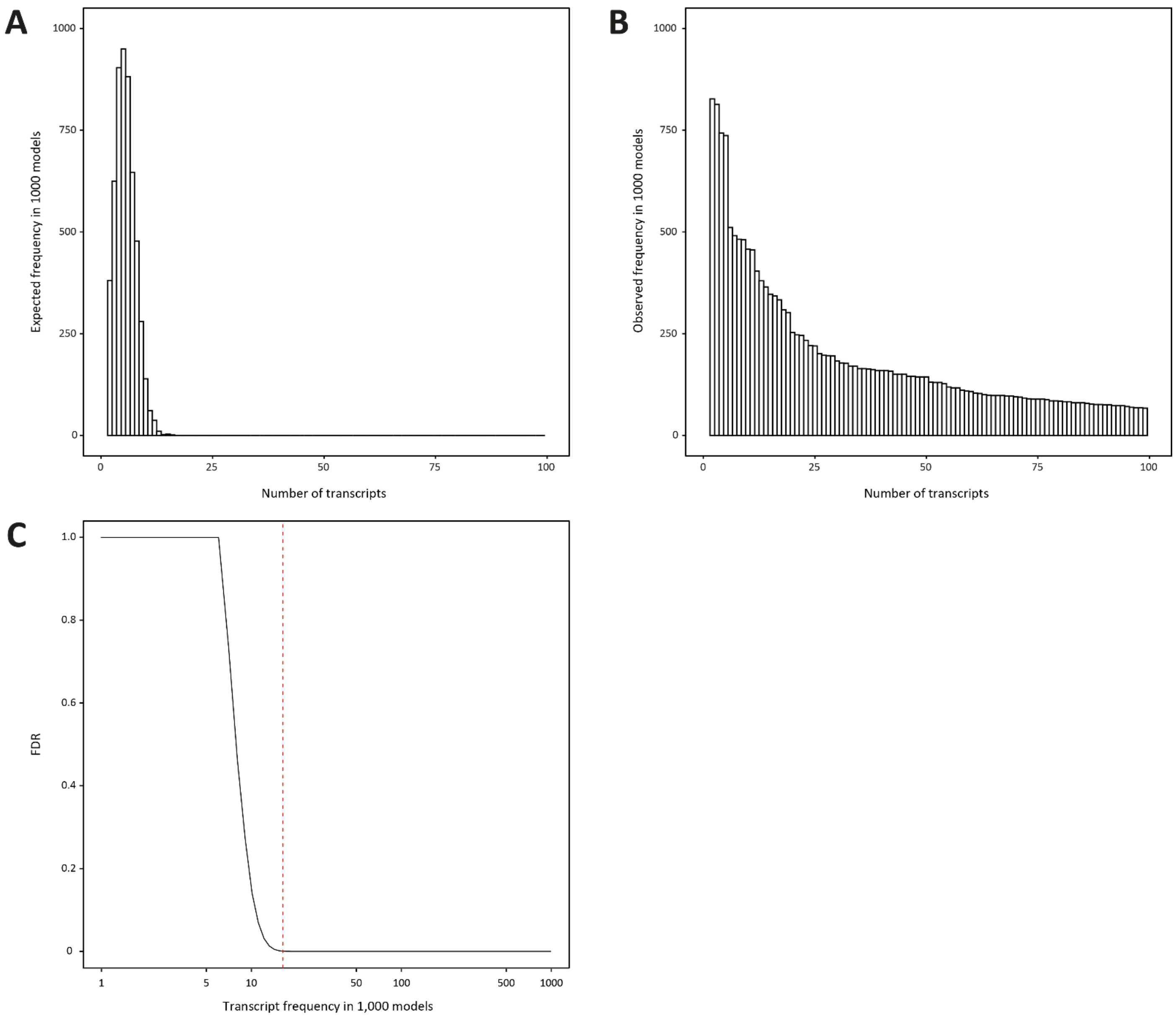

Supplemental Figure 4 A: Histogram showing the expected frequency of transcripts in 1,000 reiterated LASSO models by chance. B: Histogram showing the observed frequency of transcripts in 1,000 LASSO models. C: Lineplot indicating the association between the FDR and the selection frequency of transcripts in 1,000 LASSO models. Vertical red line is set at frequency of 16 corresponding to FDR < 0.001. LASSO = least absolute shrinkage and selection operator; FDR = false discovery rate.

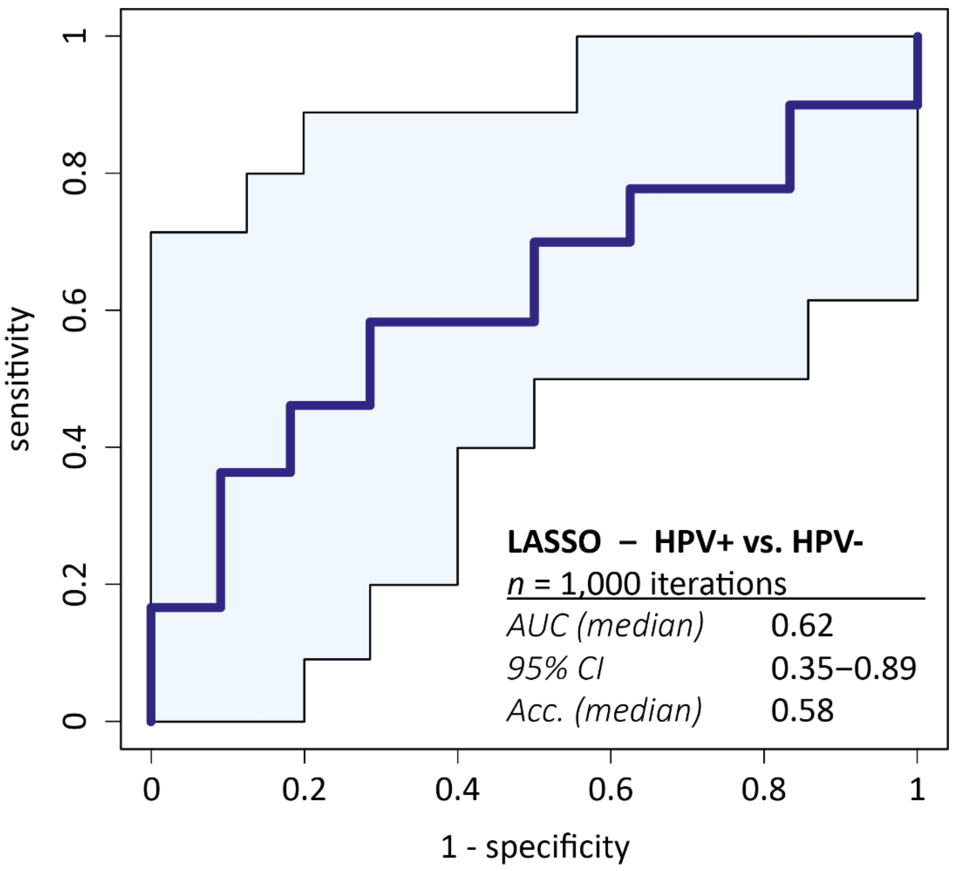

Supplemental Figure 5 ROC curve and summary of performance of validation series for 1,000 LASSO models on the dataset of HPV- (*n* = 25) and HPV+ OPSCC (*n* = 30). ROC = receiver operator characteristic; LASSO = least absolute shrinkage and selection operator; HNSCC = head and neck squamous cell carcinoma; HPV = human papillomavirus; AUC = area under the curve; CI = confidence interval; acc. = accuracy.

SVM-1			SVM-2		SVM-3		LASSO			
ENSG00000173020	ENSG00000138593	ENSG00000110768	ENSG00000137500	ENSG00000148634	ENSG00000142208	ENSG00000160691	ENSG00000142208	ENSG00000184900	ENSG00000184640	ENSG00000107077
ENSG00000242588	ENSG00000114805	ENSG00000211450	ENSG00000054282	ENSG00000143119	ENSG00000140471	ENSG00000155657	ENSG00000116473	ENSG00000140396	ENSG00000108349	ENSG00000134996
ENSG00000108654	ENSG00000163719	ENSG00000160299	ENSG00000108264	ENSG00000110717	ENSG00000128563	ENSG00000182179	ENSG00000108100	ENSG00000166925	ENSG00000127054	ENSG00000149531
ENSG00000100364	ENSG00000144048	ENSG00000213619	ENSG00000128563	ENSG00000090316	ENSG00000116473	ENSG00000124207	ENSG00000238683	ENSG00000173559	ENSG00000216863	ENSG00000185015
ENSG00000142208	ENSG00000148834	ENSG00000175575	ENSG00000146872	ENSG00000160695	ENSG00000109452	ENSG00000064933	ENSG00000134046	ENSG00000101639	ENSG00000240065	ENSG00000198363
ENSG00000101470	ENSG00000065518	ENSG00000064313	ENSG00000173020	ENSG00000114573	ENSG00000053254	ENSG00000106105	ENSG00000243955	ENSG00000147403	ENSG00000003056	ENSG00000038274
ENSG00000164733	ENSG00000186283	ENSG00000176444	ENSG00000108100	ENSG00000145349	ENSG00000166266	ENSG00000147533	ENSG00000145349	ENSG00000185215	ENSG00000015475	ENSG00000104412
ENSG00000197386	ENSG00000108518	ENSG00000107021	ENSG00000166266	ENSG00000123908	ENSG00000134046	ENSG00000065615	ENSG00000173020	ENSG00000085231	ENSG00000130748	ENSG00000105926
ENSG00000108264	ENSG00000100258	ENSG00000101224	ENSG00000100364	ENSG00000105372	ENSG00000175294	ENSG00000100325	ENSG00000128989	ENSG00000068831	ENSG00000136929	ENSG00000113282
ENSG00000163534	ENSG00000251301	ENSG00000146223	ENSG00000160551	ENSG00000167613	ENSG00000072274	ENSG00000160584	ENSG00000100364	ENSG00000147853	ENSG00000153774	ENSG00000131725
ENSG00000125304	ENSG00000229124	ENSG00000108591	ENSG00000103479	ENSG00000146247	ENSG00000071564	ENSG00000153574	ENSG00000173598	ENSG00000175606	ENSG00000168439	ENSG00000132341
ENSG00000109320	ENSG00000161533	ENSG00000127824	ENSG00000140471	ENSG00000084463	ENSG00000110717	ENSG00000167984	ENSG00000140105	ENSG00000160691	ENSG00000100181	ENSG00000134825
ENSG00000109390	ENSG00000169896	ENSG00000198851	ENSG00000063978	ENSG00000103184	ENSG00000104872	ENSG00000156471	ENSG00000153147	ENSG00000012983	ENSG00000101558	ENSG00000140682
ENSG00000127022	ENSG00000100412	ENSG00000100731	ENSG00000140564	ENSG00000126777	ENSG00000108100	ENSG00000203875	ENSG00000068383	ENSG00000164120	ENSG00000124126	ENSG00000153347
ENSG00000147894	ENSG00000074696	ENSG00000149179	ENSG00000027697	ENSG00000171307	ENSG00000108021	ENSG00000064652	ENSG00000123144	ENSG00000100796	ENSG00000143641	ENSG00000159339
ENSG00000004799	ENSG00000241973	ENSG00000178950	ENSG00000163527	ENSG00000136937	ENSG00000116514	ENSG00000128383	ENSG00000257261	ENSG00000105829	ENSG00000188612	ENSG00000168765
ENSG00000188186	ENSG00000133317	ENSG00000196352	ENSG00000124275	ENSG00000143401	ENSG00000153914	ENSG00000006015	ENSG00000134440	ENSG00000011132	ENSG00000255028	
ENSG00000170581	ENSG00000197265	ENSG00000044115	ENSG00000104853	ENSG00000170322	ENSG00000027697	ENSG00000113522	ENSG00000141552	ENSG00000131188	ENSG00000068784	
ENSG00000223501	ENSG00000112339	ENSG00000104886	ENSG00000163359	ENSG00000115457	ENSG00000046651	ENSG00000162434	ENSG00000257207	ENSG00000109062	ENSG00000124795	
ENSG00000071564	ENSG00000135124	ENSG00000100353	ENSG00000153914	ENSG00000115368	ENSG00000141480	ENSG00000179364	ENSG00000146535	ENSG00000079805	ENSG00000147535	
ENSG00000048649	ENSG00000166197	ENSG00000107862	ENSG00000090861	ENSG00000177868	ENSG00000071537	ENSG00000137845	ENSG00000206052	ENSG00000130725	ENSG00000166946	
ENSG00000204713	ENSG00000153574	ENSG00000148175	ENSG00000134046	ENSG00000184588	ENSG00000137815	ENSG00000113282	ENSG00000172037	ENSG00000036257	ENSG00000175029	
ENSG00000108100	ENSG00000168906	ENSG00000136021	ENSG00000133112	ENSG00000116199	ENSG00000152404	ENSG00000110851	ENSG00000136824	ENSG00000068323	ENSG00000198771	
ENSG00000103479	ENSG00000058272	ENSG00000116898	ENSG00000128604	ENSG00000133193	ENSG00000159377	ENSG00000145901	ENSG00000115944	ENSG00000183576	ENSG00000021574	
ENSG00000171634	ENSG00000105223	ENSG00000077420	ENSG00000231925	ENSG00000196187	ENSG00000178971	ENSG00000103275	ENSG00000111817	ENSG00000103194	ENSG00000100380	
ENSG00000156411	ENSG00000088682	ENSG00000103184	ENSG00000044574	ENSG00000132591	ENSG00000125835	ENSG00000007168	ENSG00000158941	ENSG00000109184	ENSG00000103479	
ENSG00000134686	ENSG00000151651	ENSG00000172819	ENSG00000166887	ENSG00000229124	ENSG00000174720	ENSG00000100412	ENSG00000135404	ENSG00000164134	ENSG00000105401	
ENSG00000197461	ENSG00000154451	ENSG00000132549	ENSG00000160813	ENSG00000140474	ENSG00000146535	ENSG00000109466	ENSG00000143622	ENSG00000230989	ENSG00000105443	
ENSG00000188404	ENSG00000133812	ENSG00000127314	ENSG00000072274	ENSG00000053900	ENSG00000159461	ENSG00000111642	ENSG00000162598	ENSG00000147099	ENSG00000136732	
ENSG00000149177	ENSG00000203666	ENSG00000007237	ENSG00000197756	ENSG00000108587	ENSG00000131788	ENSG00000138834	ENSG00000211896	ENSG00000198818	ENSG00000155926	
ENSG00000105887	ENSG00000165271	ENSG00000147443	ENSG00000100280	ENSG00000114391	ENSG00000264538	ENSG00000137075	ENSG00000105048	ENSG00000103365	ENSG00000180879	
ENSG00000244038	ENSG00000160932	ENSG00000114942	ENSG00000136653	ENSG00000113441	ENSG00000138119	ENSG00000120029	ENSG00000100079	ENSG00000115271	ENSG00000204136	
ENSG00000090861	ENSG00000116741	ENSG00000236279	ENSG00000135018	ENSG00000187667	ENSG00000166734	ENSG00000166200	ENSG00000107771	ENSG00000254810	ENSG00000085491	
ENSG00000123144	ENSG00000100029	ENSG00000172164	ENSG00000183011	ENSG00000104957	ENSG00000182606	ENSG00000122008	ENSG00000249307	ENSG00000158517	ENSG00000117115	
ENSG00000128563	ENSG00000171566	ENSG00000197150	ENSG00000183621	ENSG00000127526	ENSG00000160813	ENSG00000088387	ENSG00000046651	ENSG00000225205	ENSG00000123505	
ENSG00000133112	ENSG00000164118	ENSG00000106244	ENSG00000109452	ENSG00000147650	ENSG00000142546	ENSG00000129003	ENSG00000114127	ENSG00000100519	ENSG00000132463	
ENSG00000167641	ENSG00000132792	ENSG00000170271	ENSG00000110075	ENSG00000134324	ENSG00000132589	ENSG00000142230	ENSG00000120705	ENSG00000227355	ENSG00000138772	
ENSG00000112697	ENSG00000198604	ENSG00000179361	ENSG00000158717	ENSG00000074800	ENSG00000169826	ENSG00000163655	ENSG00000264538	ENSG00000065243	ENSG00000169490	
ENSG00000100362	ENSG00000151465	ENSG00000104731	ENSG00000177189	ENSG00000087365	ENSG00000049656	ENSG00000117533	ENSG00000119421	ENSG00000163444	ENSG00000177666	
ENSG00000198218	ENSG00000144554	ENSG00000166037	ENSG00000071553	ENSG00000083535	ENSG00000197746	ENSG00000181036	ENSG00000139505	ENSG00000119487	ENSG00000272888	
ENSG00000104974	ENSG00000181163	ENSG00000107938	ENSG00000187742	ENSG00000066135	ENSG00000110048	ENSG00000084072	ENSG00000184076	ENSG00000131711	ENSG00000087074	
ENSG00000118900	ENSG00000134644	ENSG00000128534	ENSG00000196405	ENSG00000230989	ENSG00000125977	ENSG00000091409	ENSG00000071564	ENSG00000166483	ENSG00000130176	
ENSG00000185359	ENSG00000079459	ENSG00000213523	ENSG00000082805	ENSG00000068354	ENSG00000249307	ENSG00000184500	ENSG00000123124	ENSG00000187742	ENSG00000132589	
ENSG00000037474	ENSG00000089693	ENSG00000138798	ENSG00000125835	ENSG00000126773	ENSG00000134851	ENSG00000119402	ENSG00000140848	ENSG00000169826	ENSG00000172354	
ENSG00000132274	ENSG00000148290	ENSG00000139722	ENSG00000132205	ENSG00000044115	ENSG00000180182	ENSG00000104969	ENSG00000153207	ENSG00000198876	ENSG00000183172	
ENSG00000115232	ENSG00000241468		ENSG00000135932	ENSG00000084070	ENSG00000141552	ENSG00000166925	ENSG00000156875	ENSG00000088038	ENSG00000223519	
ENSG00000084754	ENSG00000109689		ENSG00000170876	ENSG00000075568	ENSG00000104974	ENSG00000196756	ENSG00000196262	ENSG00000108010	ENSG00000229124	
ENSG00000138413	ENSG00000165487		ENSG00000054983	ENSG00000171490	ENSG00000177189	ENSG00000119421	ENSG00000113615	ENSG00000126934	ENSG00000231925	
ENSG00000125755	ENSG00000146731		ENSG00000182899	ENSG00000145246	ENSG00000140479	ENSG00000125898	ENSG00000154723	ENSG00000155158	ENSG00000075790	
ENSG00000158578	ENSG00000173992		ENSG00000067955	ENSG00000198168	ENSG00000159692	ENSG00000131389	ENSG00000186298	ENSG00000197459	ENSG00000129103	
ENSG00000142687	ENSG00000151883		ENSG00000102595	ENSG00000134014	ENSG00000065970	ENSG00000112081	ENSG00000154359	ENSG00000002330	ENSG00000134308	
ENSG00000198648	ENSG00000117614		ENSG00000084090	ENSG00000150054	ENSG00000173598	ENSG00000169375	ENSG00000103642	ENSG00000071243	ENSG00000142347	
ENSG00000020633	ENSG00000142949		ENSG00000059378	ENSG00000136811	ENSG00000174444	ENSG00000076554	ENSG00000233968	ENSG00000110013	ENSG00000148218	
ENSG00000064012	ENSG00000111231		ENSG00000158850	ENSG00000010292	ENSG00000100280	ENSG00000160201	ENSG00000109390	ENSG00000139835	ENSG00000158201	
ENSG00000105383	ENSG00000108061		ENSG00000168394	ENSG00000125870	ENSG00000103249	ENSG00000093000	ENSG00000122122	ENSG00000198805	ENSG00000255240	
ENSG00000142227	ENSG00000164171		ENSG00000012660	ENSG00000167552	ENSG00000137804	ENSG00000005022	ENSG00000111554	ENSG00000141068	ENSG00000011007	
ENSG00000077147	ENSG00000121749		ENSG00000221983	ENSG00000154146	ENSG00000148634	ENSG00000028528	ENSG00000149243	ENSG00000143933	ENSG00000092841	
ENSG00000138660	ENSG00000181036		ENSG00000086730	ENSG00000055211	ENSG00000110075	ENSG00000100239	ENSG00000196407	ENSG00000182463	ENSG00000115415	
ENSG00000094880	ENSG00000131171		ENSG00000156136	ENSG00000151923	ENSG00000078369	ENSG00000146463	ENSG00000175984	ENSG00000185651	ENSG00000141127	
ENSG00000138758	ENSG00000228651		ENSG00000204634	ENSG00000150093	ENSG00000120705	ENSG00000112062	ENSG00000013810	ENSG00000204710	ENSG00000166579	
ENSG00000158863	ENSG00000093167		ENSG00000175224	ENSG00000164754	ENSG00000106348	ENSG00000177054	ENSG00000109790	ENSG00000048649	ENSG00000169764	
ENSG00000120675	ENSG00000174437		ENSG00000046651	ENSG00000100519	ENSG00000177728	ENSG00000170866	ENSG00000117151	ENSG00000121210	ENSG00000172992	
ENSG00000131788	ENSG00000124783		ENSG00000145494	ENSG00000175115	ENSG00000005007	ENSG00000121104	ENSG00000002549	ENSG00000251301	ENSG00000175220	
ENSG00000085377	ENSG00000066455		ENSG00000182087	ENSG00000241837	ENSG00000111647	ENSG00000138795	ENSG00000141867	ENSG00000023892	ENSG00000222041	
ENSG00000111335	ENSG00000071794		ENSG00000196562	ENSG00000136754	ENSG00000109184	ENSG00000144635	ENSG00000144744	ENSG00000135919	ENSG00000069943	
ENSG00000165525	ENSG00000225205		ENSG00000106524	ENSG00000213339	ENSG00000133275	ENSG00000168159	ENSG00000170315	ENSG00000059378	ENSG00000072506	
ENSG00000070831	ENSG00000100504		ENSG00000153575	ENSG00000164081	ENSG00000151461	ENSG00000241837	ENSG00000177868	ENSG00000085719	ENSG00000088832	
ENSG00000128989	ENSG00000113593		ENSG00000155657	ENSG00000156873	ENSG00000068784	ENSG00000100726	ENSG00000073578	ENSG00000096060	ENSG00000115825	
ENSG00000116251	ENSG00000233093		ENSG00000072958	ENSG00000117533	ENSG00000112079	ENSG00000105325	ENSG00000142327	ENSG00000099804	ENSG00000125970	
ENSG00000082014	ENSG00000103005		ENSG00000198848	ENSG00000059377	ENSG00000145740	ENSG00000139726	ENSG00000198948	ENSG00000134056	ENSG00000139433	
ENSG00000178927	ENSG00000100284		ENSG00000163513	ENSG00000136527	ENSG00000185624	ENSG00000145741	ENSG00000156411	ENSG00000146833	ENSG00000139842	
ENSG00000171608	ENSG00000089063		ENSG00000168172	ENSG00000205639	ENSG00000136108	ENSG00000182149	ENSG00000204632	ENSG00000153815	ENSG00000142949	
ENSG00000119977	ENSG00000130023		ENSG00000102901	ENSG00000197136	ENSG00000198728	ENSG00000064201	ENSG00000140264	ENSG00000163041	ENSG00000147687	
ENSG00000163359	ENSG00000121858		ENSG00000143612	ENSG00000117632	ENSG00000123066	ENSG00000101365	ENSG00000128585	ENSG00000174444	ENSG00000160712	
ENSG00000160255	ENSG00000166411		ENSG00000159335	ENSG00000166091	ENSG00000143324	ENSG00000116906	ENSG00000182179	ENSG00000177981	ENSG00000170144	
ENSG00000140575	ENSG00000048544		ENSG00000177410	ENSG00000104814	ENSG00000102978	ENSG00000133398	ENSG00000248334	ENSG00000184203	ENSG00000070785	
ENSG00000213614	ENSG00000021355		ENSG00000172667	ENSG00000140368	ENSG00000168894	ENSG00000140598	ENSG00000131165	ENSG00000196233	ENSG00000091527	
ENSG00000172795	ENSG00000184900		ENSG00000107175	ENSG00000096968	ENSG00000159459	ENSG00000172500	ENSG00000107736	ENSG00000198924	ENSG00000099940	
ENSG00000109775	ENSG00000188612		ENSG00000051108	ENSG00000110395	ENSG00000105323	ENSG00000127527	ENSG00000164733	ENSG00000109452	ENSG00000101079	
ENSG00000137076	ENSG00000186625		ENSG00000172340	ENSG00000177666	ENSG00000106829	ENSG00000073050	ENSG00000268555	ENSG00000118564	ENSG00000120675	
ENSG00000111644	ENSG00000148180		ENSG00000148303	ENSG00000128815	ENSG00000122557	ENSG00000137478	ENSG00000148672	ENSG00000131100	ENSG00000134201	
ENSG00000170542	ENSG00000135842		ENSG00000142864	ENSG00000214941	ENSG00000140386	ENSG00000167522	ENSG00000164111	ENSG00000149923	ENSG00000145740	
ENSG00000166825	ENSG00000099968		ENSG00000198818	ENSG00000139946	ENSG00000159625	ENSG00000115548	ENSG00000075785	ENSG00000172572	ENSG00000145982	
ENSG00000178234	ENSG00000012124		ENSG00000233968	ENSG00000177981	ENSG00000172262	ENSG00000143933	ENSG00000168159	ENSG00000070831	ENSG00000170365	
ENSG00000117335	ENSG00000198924		ENSG00000111196	ENSG00000149531	ENSG00000091157	ENSG00000172977	ENSG00000186480	ENSG00000084112	ENSG00000175470	
ENSG00000104998	ENSG00000205038		ENSG00000220804	ENSG00000163162	ENSG00000126088	ENSG00000065911	ENSG00000147416	ENSG00000100811	ENSG00000178741	
ENSG00000038358	ENSG00000152229		ENSG00000107863	ENSG00000196914	ENSG00000101849	ENSG00000138107	ENSG00000007255	ENSG00000112303	ENSG00000233016	
ENSG00000023318	ENSG00000138442		ENSG00000122203	ENSG00000172819	ENSG00000213639	ENSG00000185015	ENSG00000095139	ENSG00000117676	ENSG00000253982	
ENSG00000084070	ENSG00000078808		ENSG00000101849	ENSG00000060491	ENSG00000087338	ENSG00000073578	ENSG00000140564	ENSG00000130755	ENSG00000062598	
ENSG00000070061	ENSG00000100938		ENSG00000088726	ENSG00000075413	ENSG00000164164	ENSG00000079482	ENSG00000084754	ENSG00000138495	ENSG00000064490	
ENSG00000136710	ENSG00000142794		ENSG00000085377	ENSG00000175470	ENSG00000102710	ENSG00000166946	ENSG00000112787	ENSG00000174799	ENSG00000115419	
ENSG00000166925	ENSG00000032742		ENSG00000129003	ENSG00000090565	ENSG00000204574	ENSG00000101972	ENSG00000105254	ENSG00000220804	ENSG00000118308	
ENSG00000116663	ENSG00000124491		ENSG00000158805	ENSG00000118454	ENSG00000198265	ENSG00000145725	ENSG00000126217	ENSG00000244509	ENSG00000129354	
ENSG00000002330	ENSG00000023191		ENSG00000107372	ENSG00000132507	ENSG00000165678	ENSG00000118418	ENSG00000198258	ENSG00000067829	ENSG00000142794	
ENSG00000140367	ENSG00000197555		ENSG00000007168	ENSG00000088833	ENSG00000175334	ENSG00000083312	ENSG00000263563	ENSG00000138801	ENSG00000151247	
ENSG00000116701	ENSG00000165119		ENSG00000143641	ENSG00000089351	ENSG00000165416	ENSG00000150054	ENSG00000213654	ENSG00000231607	ENSG00000161533	
ENSG00000126768	ENSG00000162654		ENSG00000101182	ENSG00000074696	ENSG00000113575	ENSG00000106537	ENSG00000100362	ENSG00000121741	ENSG00000164054	
ENSG00000130309	ENSG00000107798		ENSG00000113013	ENSG00000111906	ENSG00000074054	ENSG00000075089	ENSG00000205542	ENSG00000129968	ENSG00000004766	
ENSG00000113810	ENSG00000164329		ENSG00000146376	ENSG00000001497	ENSG00000163512	ENSG00000106244	ENSG00000122694	ENSG00000160551	ENSG00000005483	
ENSG00000128383	ENSG00000070610		ENSG00000129187	ENSG00000149231	ENSG00000153214	ENSG00000107949	ENSG00000089053	ENSG00000163719	ENSG00000102974	

Supplemental Table 1 Overview of selected biomarker panels.

GO term ID	Term description	Observed gene count	Background gene count	Strength	Signal	False discovery rate
GO:0008152	Metabolic process	169	7988	0.16	0.54	4.30e-06
GO:1901564	Organonitrogen compound metabolic process	117	4981	0.21	0.53	5.60e-05
GO:0071704	Organic substance metabolic process	156	7522	0.15	0.48	0.00010
GO:0044238	Primary metabolic process	149	7156	0.16	0.46	0.00019
GO:0051726	Regulation of cell cycle	40	1108	0.4	0.56	0.00053
GO:0006807	Nitrogen compound metabolic process	138	6643	0.16	0.42	0.00079
GO:0044237	Cellular metabolic process	137	6568	0.16	0.43	0.00079
GO:0010564	Regulation of cell cycle process	29	716	0.45	0.51	0.0020
GO:0044770	Cell cycle phase transition	14	183	0.72	0.6	0.0020
GO:0044772	Mitotic cell cycle phase transition	13	173	0.71	0.53	0.0046
GO:0019538	Protein metabolic process	89	3910	0.2	0.36	0.0071
GO:0015980	Energy derivation by oxidation of organic compounds	15	262	0.6	0.42	0.0149
GO:0021762	Substantia nigra development	7	47	1.01	0.47	0.0149
GO:0006091	Generation of precursor metabolites and energy	19	411	0.5	0.39	0.0167
GO:0007049	Cell cycle	38	1246	0.32	0.35	0.0167
GO:0050790	Regulation of catalytic activity	60	2370	0.24	0.33	0.0167
GO:0006796	Phosphate-containing compound metabolic process	50	1877	0.26	0.32	0.0230
GO:0009987	Cellular process	244	14826	0.05	0.27	0.0230
GO:0009060	Aerobic respiration	11	161	0.67	0.37	0.0301
GO:0043170	Macromolecule metabolic process	116	5781	0.14	0.28	0.0301
GO:0051656	Establishment of organelle localization	17	380	0.49	0.31	0.0453

Supplemental Table 2 STRING analyses output: summary of 21 biological processes enriched for (FDR < 0.05) in 316 transcripts most frequently selected in 1,000 LASSO iterations.
